# Infected Dendritic Cells Facilitate Systemic Dissemination and Transplacental Passage of the Obligate Intracellular Parasite *Neospora caninum* in Mice

**DOI:** 10.1371/journal.pone.0032123

**Published:** 2012-03-05

**Authors:** Esther Collantes-Fernandez, Romanico B. G. Arrighi, Gema Álvarez-García, Jessica M. Weidner, Javier Regidor-Cerrillo, John C. Boothroyd, Luis M. Ortega-Mora, Antonio Barragan

**Affiliations:** 1 Center for Infectious Medicine, Department of Medicine, Karolinska Institutet, Stockholm, Sweden; 2 Swedish Institute for Communicable Disease Control, Stockholm, Sweden; 3 SALUVET, Animal Health Department, Faculty of Veterinary Sciences, Complutense University of Madrid, Madrid, Spain; 4 Department of Microbiology and Immunology, Stanford University School of Medicine, Stanford, California, United States of America; University of Oklahoma Health Sciences Center, United States of America

## Abstract

The obligate intracellular parasite *Neospora caninum* disseminates across the placenta and the blood-brain barrier, to reach sites where it causes severe pathology or establishes chronic persistent infections. The mechanisms used by *N. caninum* to breach restrictive biological barriers remain elusive. To examine the cellular basis of these processes, migration of different *N. caninum* isolates (Nc-1, Nc-Liverpool, Nc-SweB1 and the Spanish isolates: Nc-Spain 3H, Nc-Spain 4H, Nc-Spain 6, Nc-Spain 7 and Nc-Spain 9) was studied in an *in vitro* model based on a placental trophoblast-derived BeWo cell line. Here, we describe that infection of dendritic cells (DC) by *N. caninum* tachyzoites potentiated translocation of parasites across polarized cellular monolayers. In addition, powered by the parasite's own gliding motility, extracellular *N. caninum* tachyzoites were able to transmigrate across cellular monolayers. Altogether, the presented data provides evidence of two putative complementary pathways utilized by *N. caninum*, in an isolate-specific fashion, for passage of restrictive cellular barriers. Interestingly, adoptive transfer of tachyzoite-infected DC in mice resulted in increased parasitic loads in various organs, e.g. the central nervous system, compared to infections with free parasites. Inoculation of pregnant mice with infected DC resulted in an accentuated vertical transmission to the offspring with increased parasitic loads and neonatal mortality. These findings reveal that *N. caninum* exploits the natural cell trafficking pathways in the host to cross cellular barriers and disseminate to deep tissues. The findings are indicative of conserved dissemination strategies among coccidian apicomplexan parasites.

## Introduction

*Neospora caninum* is a single-celled coccidian parasite belonging to the apicomplexan phylum and closely related to *Toxoplasma gondii*. *N. caninum* has emerged as an important cause of reproductive failure in cattle worldwide, leading to significant economic losses in beef and dairy cattle industries [1]. Infection in cattle may occur by horizontal transmission when cattle ingest sporulated oocysts shed by a canid definitive host, or by endogenous congenital transmission from a persistently infected dam to a fetus [2]. Primary infection or recrudescent infection in a pregnant cow can result in abortion, birth of a weak calf or birth of a clinically healthy but persistently infected calf [3], [4].

*N. caninum* is one of the most efficiently transplacentally-transmitted organisms in cattle [2]. During natural infections, *N. caninum* disseminates widely and crosses the placenta and the blood-brain barrier. There, it may cause severe acute pathology or establish chronic infections, e.g. in the developing fetus or in the central nervous system (CNS). The mechanisms by which *N. caninum* manages systemic dissemination and breaches restrictive biological barriers remain unknown. Previous studies on *T. gondii* have demonstrated that extracellular tachyzoite stages are able to actively cross polarized cell monolayers and that this ability is linked to parasite gliding motility and virulence [5], [6]. Alternatively, parasites may exploit immune host cells that display basal or rapidly inducible motile properties to disseminate. In this context, dendritic cells (DC) have recently been identified as systemic carriers (Trojan horses) of *T. gondii* tachyzoites; the parasite induces a migratory phenotype in DC that potentiates parasite dissemination in a strain type specific manner [7], [8], [9], [10]. *N. caninum* can infect different immune cell types including DC, macrophages and natural killer (NK) cells [11], [12], [13] in a similar fashion as *T. gondii* [14]. Recently, mouse bone-marrow derived DC were shown to be permissive to invasion and replication by *N. caninum* [12], [13]. The placenta plays a key role in the pathogenesis of neosporosis. Yet, the mechanisms by which *N. caninum* crosses the placental barrier are largely unknown. Maternal leukocytes do not routinely traffic across the placenta [15], thus parasite active motility could represent a mode for *N. caninum* to traverse the non-permissive placenta barrier. However, there is also mounting evidence for the regulated presence of leukocytes at the fetal-maternal interface during pregnancy and pathologic conditions may facilitate or induce the transfer of maternal cells [16].

Recent findings indicate that there are wide pathogenic and vertical transmission variabilities among *N. caninum* isolates [17], [18] and a vast genetic diversity among isolates [19]. Yet, the determinants of transplacental passage for different isolates remain unknown and specific genetic markers for virulent traits remain unidentified.

Here, we investigate the migratory pathways utilized by *N. caninum* using *in vitro* and *in vivo* models of infection. We establish that *N. caninum* migrates across polarized trophoblastic monolayers powered by the parasite's own active gliding motility and that infected DC translocate parasites across polarized trophoblastic monolayers *in vitro*. Furthermore, adoptive transfer of *Neospora*-infected DC in mice led to higher parasite tissue burdens, earlier parasite presence in the CNS and promoted *N. caninum* vertical transmission. Altogether, the presented data provides evidence of two putative complementary pathways utilized by *N. caninum* for dissemination and transplacental passage.

## Materials and Methods

### Ethical statement

All protocols involving animals were approved by the Regional Animal Research Ethical Board, Stockholm, Sweden (N250/09, N215/09, N223/09) and the Animal Research Committee of the Complutense University, Madrid, Spain, following proceedings described in Regulation of Internal Regime for Animal Research Committee (published at BOUC, no. 2, at 9 February 2006) and EU legislation (Council Directive 86/609/EEC). The Regional Ethics Committee, Stockholm, Sweden, approved protocols involving human cells (2006/116-31). All donors received written and oral information upon donation of blood at the Karolinska University Hospital. Written consent was obtained for utilization of white blood cells for research purposes. The ethics committees approved this consent procedure.

### Parasites, cell lines and mice

*N. caninum* isolates included Nc-1 [20], Nc-Liverpool [21], Nc-SweB1 [22] and the Spanish isolates: Nc-Spain 3H, Nc-Spain 4H, Nc-Spain 6, Nc-Spain 7 and Nc-Spain 9 [23]. *T. gondii* strains used include green fluorescent protein (GFP)-expressing lines RH-LDM [6] (cloned from RH-GFPS65T [24]) and ME49-PTG (PTG-GFPS65T [24]). All parasites were maintained by serial passage in human foreskin fibroblast (HFF) monolayers cultured in Dulbecco modified Eagle medium (DMEM, Invitrogen) with 10% fetal calf serum, 20 µg/ml gentamicin, 2 mM L-glutamine, and 0.01 M HEPES, referred to as complete medium (CM) [7]. The polarizing BeWo choriocarcinoma cell line was obtained from Dr. A. Schwartz, Washington University School of Medicine, St. Louis, MO [25] and maintained in CM.

BALB/c mice were obtained from the Comparative Medicine Facility, Karolinska Institutet or from a commercial supplier (Harlan Interfauna Ibérica).

### Generation of luciferase-expressing *N. caninum* isolate

Luciferase-expressing *N. caninum* (Nc-1*Luc*) was used to visualize *N. caninum* infection by bioluminescence imaging in mice. A clonal HXGPRT-deficient mutant of *N. caninum* (Nc-1 *hxgprt*-), obtained by chemical mutagenesis and selection for resistance to 6-thioxanthine as previously described for *T. gondii* [26] was employed to constitutively express the firefly luciferase protein in the tachyzoite stage following previous procedures [26]. The HXGPRT gene was cloned in between the promoter and the 5′ untranslated region (UTR) of the *T. gondii* gene for dihydrofolate reductase (DHFR) and F*luc* gene was driven by TUB1 promoter and followed by the 5′UTR in the reverse orientation of HXGPRT gene [27]. The construct was amplified and purified by means of maxiprep from previously transformed *E. coli*. Next, restriction enzyme-mediated integration was applied to optimize the plasmid insertion rate in the genome. Fifty micrograms of plasmid DNA was linearized with *Sac*I and transfected by electroporation in the presence of *Sac*I into 10^7^ freshly harvested Nc-1 *hxgprt*- tachyzoites, as previously described [28], [29]. After 24 h, stably transfected tachyzoites were selected with mycophenolic acid and xanthine (50 µg/ml each) (Sigma), and drug-resistant clones were obtained from independent populations by limiting dilution. All selected resistant clones proliferated in a similar manner and produced similar levels of light when D-luciferin potassium salt (Xenogen) was added (0.15 mg/ml) to freshly purified tachyzoites. Ten fold diluted tachyzoites (10^4^, 10^5^ and 10^6^) were resuspended in 100 µl of cell culture medium and 10 µl of D-luciferin (0.15 mg/ml) and incubated for 10 min at 37°C. The light emitted was measured in a Monolight 2010 luminometer (Analytical Luminescence Laboratory) as relative light units (RLU). RLU values for 10^4^, 10^5^ and 10^6^ tachyzoites were 760, 6944 and 68433, respectively. The genetic manipulation necessary to express luciferase in the Nc-1 isolate did not result in any significant alterations in the behaviour of the organisms either *in vivo* or *in vitro*.

### *In vitro* generated DC

Buffy coats from healthy human blood donors were treated with a monocyte enrichment cocktail (RosetteSep; StemCell Technologies), followed by centrifugation on Lymphoprep (Axis-Shield PoC AS). Monocyte-derived DC were generated as previously described [7]. The Regional Ethics Committee, Stockholm, Sweden, approved all protocols involving human cells (2006/116-31). Murine bone marrow-derived DC (BMDC) were isolated and cultured as previously described [7]. Briefly, cells from bone marrow of BALB/c mice were grown in DMEM with 10% heat-inactivated bovine fetal serum, 20 µg/ml gentamicin, 2 mM L-glutamine, 0.01 M HEPES and 10% supernatant from the GM-CSF-secreting cell line X63 [30].

### *In vitro* migration assays

Infection of DC by freshly egressed tachyzoites of *N. caninum* or *T. gondii* and quantification of migrated cells were conducted as previously described [7]. For transmigration assays, DC were plated at a density of 3×10^6^ cells/well (24-well plate) and incubated for 6 h at 37°C-5% CO_2_ with tachyzoites at the indicated multiplicity of infection (MOI), CM (non-infected DC), LPS (100 ng/ml, Sigma) or heat-inactivated tachyzoites (Nc-1 or ME49-PTG). Cells were transferred into polyester Transwell inserts (24-well plate, pore size 8 µm, BD Biosciences) and incubated overnight at 37°C-5% CO_2_. Migrated cells were quantified in a hematocytometer as previously described [7]. The frequency of transmigration was defined as the ratio between the number of transmigrated cells and the total amount of cells added.

For transmigration assays across polarized trophoblastic monolayers, BeWo cells were seeded at a density of 10^5^ cells/cm^2^ onto Transwell inserts (BD Biosciences) and grown for 5–6 d to form a polarized monolayer (resistance >2000 Ω/cm^2^ using an Ohmmeter; Millipore). To assess the functionality of the cellular barrier, FITC-Dextran (FD-100, Sigma) was added to the upper well of the Transwell system. Plates were incubated for 30 min at 37°C and fluorometric quantification (Xenogen IVIS Spectrum, 465 nm excitation and 520 nm emission) was performed in the lower well of the Transwell system. Quantification of parasite transmigration across BeWo polarized monolayers was performed by plaque counting of transmigrated parasites onto HFF monolayers seeded in the lower chamber of the Transwell system or by real-time PCR.

For assays involving tachyzoite-infected DC from various isolates, the inoculum size was normalized, taking into account the viability of the DC and the intracellular parasites, DC infection frequency and the number of parasites/infected cell as described [7], [8]. Briefly, cells were sequentially washed (80× *g* for 10 min) to remove extracellular parasites (<4%) and to enrich them for intracellular parasites (>95%). The infection rate and average number of parasites/infected cell was determined by immunofluorescence staining. Trypan blue staining was used to determine host cell and parasite viability. Additionally, parasite viability and infection frequency was determined by plaquing assays (colony-forming units -cfu- per added cell).

### DNA extraction and quantitative real-time PCR

Genomic DNA was extracted from cellular samples using the commercial kit Wizard® Genomic DNA Purification Kit (Promega) following manufacturer's instructions. DNA from mouse brains was isolated using the Maxwell robotic system by the tissue DNA extraction kit (Promega) according to the manufacturer's instruction. *N. caninum* DNA was measured by a real-time PCR using SYBR Green I, as described [31]. Parasite number in tissue samples (parasite load) was expressed as parasite number/µg host DNA.

### Plaquing assays

The number of viable parasites was determined by plaque formation on HFF monolayers as described [32]. For *N. caninum* assays, HFF monolayers were fixed in 4% paraformaldehyde, after washing, the samples were permeabilized with 0.25% Triton X-100 and blocked with PBS/3% BSA for 30 min. Parasites were then labelled with a hyperimmune rabbit antiserum that was directed against *N. caninum* tachyzoites as described previously [33]. Goat anti-rabbit conjugated to Alexa 488 (Molecular Probes) was used as secondary antibody.

### Immunofluorescence staining

DC were plated on poly-L-lysine-coated glass coverslips and infected with *N. caninum* at the indicated MOI and time periods. The cells were then washed once with BRB80 buffer [80 mM piperazine-N,N′-bis (2-ethanesulfonic acid) (PIPES), pH 6.9; 1 mM MgCl_2_; 1 mM EGTA] and then fixed with 0.3% glutaraldehyde in BRB80 for 10 min at room temperature. The cells were permeablized with 1% Triton X-100 in PBS for 15 min at room temperature. Following a brief wash with PBS, the coverslips were treated with 1 mg/ml sodium borohydride in PBS three times for 5 min each. The coverslips were blocked with PBS/3% BSA for 30 min, and parasites were then labelled with a hyperimmune rabbit antiserum that was directed against *N. caninum* tachyzoites as described previously [33]. Goat anti-rabbit conjugated to Alexa 488 (Molecular Probes) was used as secondary antibody. The cells were also incubated with phalloidin-Alexa 594 (Invitrogen), which was included in the secondary antibody step. Coverslips were mounted with DAPI (Vector Laboratories) and assessed by epifluorescence microscopy (Leica, DMRB).

### Gliding assays

Glass coverslips were coated with 50% fetal bovine serum in PBS for 1 h at 37°C as described previously [6]. Freshly harvested *N. caninum* tachyzoites were added to precoated glass coverslips and incubated at 37°C-5°CO_2_ for 40 min. The slides were briefly rinsed and fixed in 4% formalin-PBS for 20 min. Trails were visualized by immunofluorescence using a monoclonal antibody against the surface *N. caninum* protein SAG1 (kindly provided by Dr. Hemphill, Bern University). Goat anti-rabbit conjugated to Alexa 488 (Molecular Probes) was used as secondary antibody. Photomicrographs were taken with a 100× oil-immersion objective on a fluorescence microscope (Nikon) connected to a digital camera. As quantification criterion, only trails with one tachyzoite associated at one end were counted. Between 10 and 15 trails were assessed per strain and the mean was calculated in each experiment (n = 3). Average trail length in parasite body lengths (approximately 7 µm) was determined.

### Adoptive transfers of DC

Seven-day-old bone marrow-derived DC were incubated with luciferase-expressing Nc-1 tachyzoites (Nc-1*Luc*) or Nc-Spain7 tachyzoites (MOI 2) for 6 h. Cells were sequentially washed (80× *g* for 10 min) to remove extracellular parasites and cell suspensions were normalized for parasite viability, host cell viability, and infection frequency as described above [7]. The total number of parasite injected in animals was confirmed by plaquing assay and real-time PCR.

### *In vivo* bioluminescence imaging (BLI)

Six-week-old male BALB/c mice were inoculated with free Nc-1*Luc* or Nc-1*Luc*-infected DC by i.p. injection. Control groups of BALB/c mice were inoculated i.p. with PBS or non-infected DC. Animals were imaged daily by BLI (IVIS Spectrum; Xenogen) until day 13 post-infection (p.i.) as previously described [7], [34]. Briefly, mice were injected i.p. with 3 mg D-luciferin potassium salt (Caliper Life Sciences) and anaesthetized with 2.3% isoflurane prior to BLI. After 10–12 min, mice were imaged in dorsal, ventral and right lateral positions and photonic emissions were assessed. Data acquisition and analysis of photonic emissions in regions of interest (ROI) were performed by using the Living Image® software 2.20.1 (Xenogen). To address whether adoptive transfer of DC promoted rapid passage across the blood-brain barrier, parasite presence was determined on day 1 p.i. Three random animals from each group were sacrificed after imaging, organs were excised, imaged *ex vivo* and brains frozen until analyzed for *N. caninum* by real-time PCR. Additionally, for detection of *N. caninum* DNA in the brain, two sets of experiments were performed (four mice per group were infected), animals were sacrified on day 1 p.i. and brains collected for parasite detection by real-time PCR.

### Infections in pregnant mice

Pregnant mice were inoculated with free Nc-Spain 7 tachyzoites or Nc-Spain 7-infected DC at mid gestation (6–10 days of pregnancy) using a BALB/c mouse model for congenital transmission, as described previously [35], [36]. Pregnant mice were injected subcutaneously to avoid the risk to inoculate the parasite *in uterus*. Briefly, seven-week-old female BALB/c mice were mated for 4 nights following synchronization of ovulation using the Whitten effect. Only confirmed pregnant mice were used for this study and allowed to carry their pregnancy to term. Neonates and dams were sacrificed with CO_2_ gas on day 30 post-partum (pp). Litter size, stillbirth, temporal evolution and rate of neonatal mortality were determined. Clinically affected mice were humanely sacrificed by CO_2_ inhalation. *N. caninum* vertical transmission rate was determined by detecting the parasite DNA in the brain of neonates by a real-time PCR.

### Statistical analysis of data

All *in vitro* assays were conducted in triplicate for each time point and performed independently three or more times. The statistic analysis was carried out using the mean corresponding to the replicates of each experiment (n≥3). Differences in the transmigration frequencies of DC and gliding distances between groups were analyzed by one-way ANOVA. Bonferroni post-test was applied to examine all possible pairwise comparisons. The Dunnet test was used to compare all groups to the “non-infected” group. For transmigration assays across polarized BeWo monolayers, data were compared by *U*-Mann Whitney or Kruskal-Wallis tests as indicated. If the analysis by Kruskal-Wallis test finds a significant difference among the groups then the groups with the largest and the smallest means are different [36]. For the *in vivo* experiments, fertility and stillborn rates were compared by Chi-square or Fisher's exact tests. Neonatal mortality was analyzed by the Kaplan-Meier survival method and the log-rank test. Parasite presence and burdens in brain were analyzed by Fisher's exact test or Student's t test, respectively. The Spearman's rank correlation coefficient (ρ) was applied to investigate the potential association between data. Statistical analysis was carried out using GraphPad Prism 5 software.

## Results

### Live *N. caninum* tachyzoites induce transmigration of DC *in vitro*

**Figure 1:**
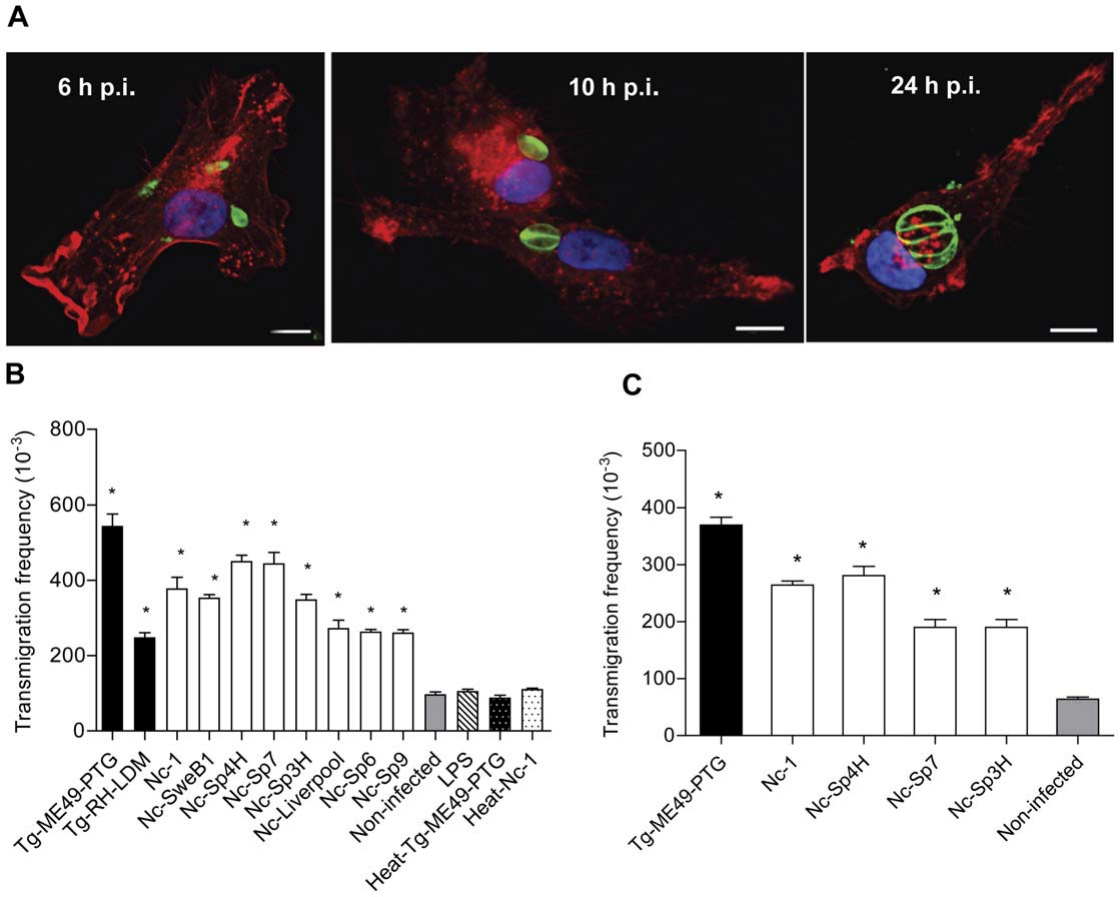
*In vitro* migratory phenotypes of DC infected with *N. caninum* and *T. gondii*. **A**. Human monocyte-derived DC are permissive to *N. caninum*. Immunofluorescence staining of DC (phalloidin-Alexa Fluor 596) infected with the isolate Nc-1*Luc* (MOI 3). Parasites were labelled with hyperimmune rabbit antiserum and Alexa Fluor 488 as secondary antibody. Overlay with DAPI (blue). Representative images of 6, 10 and 24 h p.i. are shown. Scale bar: 10 µm. **B**. Bar diagram shows the transmigration frequencies of human monocytic-derived DC incubated with live tachyzoites (MOI 2) from *T. gondii* (Tg-RH-LDM and Tg-ME49-PTG strains) and *N. caninum* (Nc-1, Nc-Liverpool, Nc-SweB1, Nc-Spain 3H, Nc-Spain 4H, Nc-Spain 6, Nc-Spain 7 and Nc-Spain 9 isolates), CM (non-infected cells), LPS (100 ng/ml) and heat-inactivated parasites (heat-Tg-ME49-PTG or heat-Nc-1) as indicated under Materials and Methods. The black bars indicate *T. gondii* and the white bars indicate *N. caninum*. Cell migration (migrated/added) was assessed by optical counting of transmigrated cells across a transwell filter. Means (±SEM) from the replicates from at least three independent experiments are shown. Asterisks indicate treatments which conferred significant differences in transmigration compared with non-infected cells (P<0.001; Dunnett's test). **C**. Transmigration of BALB/c bone marrow-derived DC infected with *T. gondii* (Tg-ME49-PTG, MOI 2) or *N. caninum* (Nc-1, Nc-Spain 4H, Nc-Spain 7 and Nc-Spain 3H, MOI 2) as indicated under Materials and Methods. Means (±SEM) from the replicates from at least three independent experiments are shown. Asterisks indicate significant differences compared to non-infected cells (P<0.05; Dunnett's test).

It was recently shown that *T. gondii* tachyzoites can induce a hypermigratory phenotype in infected DC [7]. To determine the capacity of *N. caninum* to induce DC migration, human monocyte-derived DC were incubated with freshly egressed *N. caninum* or *T. gondii* tachyzoites and allowed to migrate across a Transwell membrane as described [7], [8]. Monitoring of infection over time showed that DC were permissive to invasion and replication by *N. caninum* (Fig. 1A). Interestingly, all *N. caninum* isolates tested induced significant transmigration of DC compared to uninfected DC (P<0.001; Dunnett's test) and significant variations in transmigration frequency were found between isolates (P<0.0001; one-way ANOVA test) (Figure 1B). The highest frequency of transmigration was observed for *T. gondii* type II (Tg-ME49-PTG) followed by Nc-Spain 4H and Nc-Spain 7, whereas the infection with type I (Tg-RH-LDM), Nc-Liverpool, Nc-Spain 6 and Nc-Spain 9 induced lower frequencies of transmigration (P<0.05-0.0001; Bonferroni's post-test; Tg-ME49-PTG *versus* Tg-RH-LDM, Nc-1, Nc-SweB1, Nc-Spain 3H, Nc-Liverpool, Nc-Spain 6 and Nc-Spain 9; Nc-Spain 4H and Nc-Spain 7 *versus* Tg-RH-LDM, Nc-Liverpool, Nc-Spain 6 and Nc-Spain 9). In contrast, challenging DC with LPS or heat-inactivated tachyzoites did not significantly enhance transmigration compared to non-infected DC (P>0.05; Dunnett's test) (Figure 1B). Infection frequency of DC at 6 h p.i. was 32.4% (SD14.62) without significant differences among *N. caninum* isolates (P>0.05; one-way ANOVA test). As previously shown for *T. gondii* [7], murine bone-marrow derived DC were permissive to *N. caninum* and exhibited a very similar migratory phenotype (Figure 1C and data not shown). Taken together, these results show that infection with *N. caninum* induces a migratory phenotype in human monocyte-derived DC and murine bone marrow-derived DC and that transmigration frequency of infected DC varies in a strain specific manner.

### Extracellular tachyzoites and tachyzoite-infected DC migrate across polarized cell monolayers in a strain-dependent fashion

**Figure 2:**
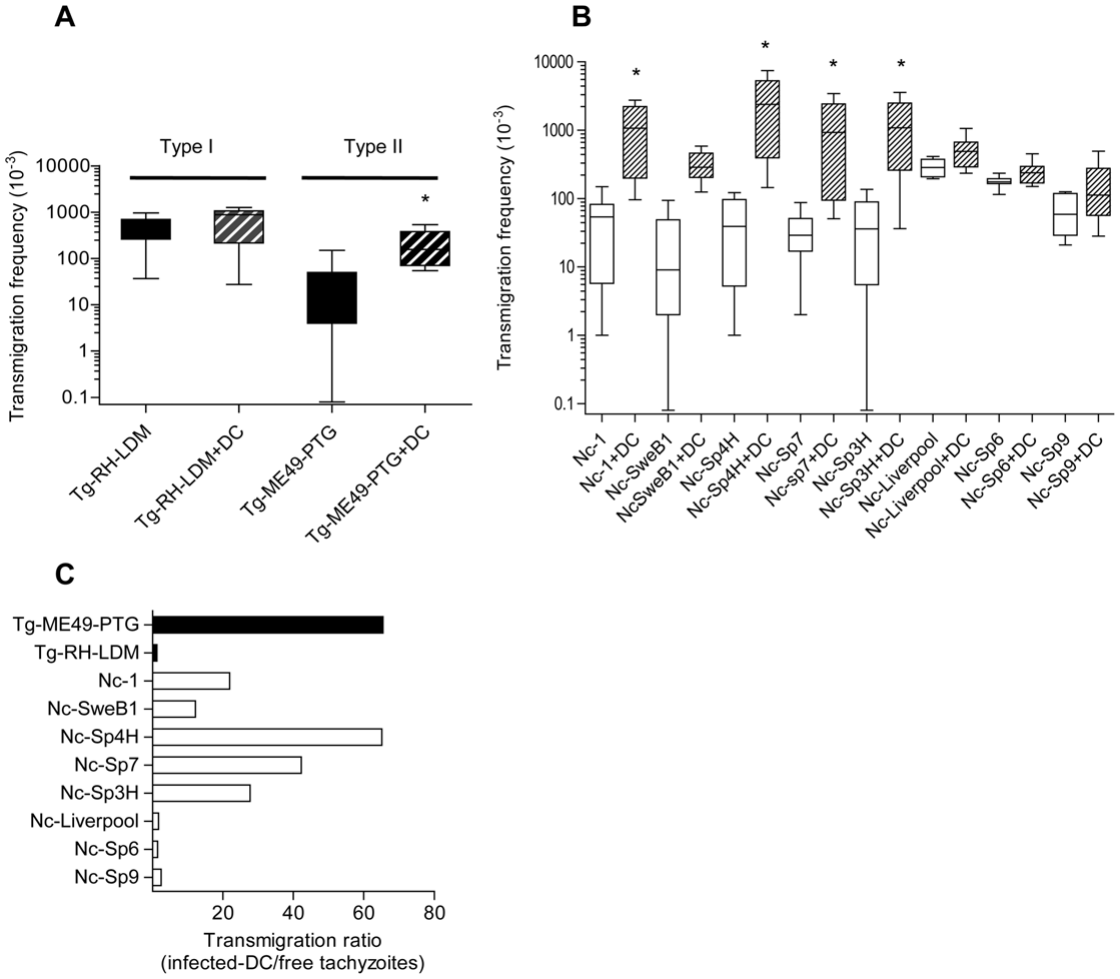
Transmigration of tachyzoites and tachyzoite-infected DC across cell monolayers. **A and B**. Transmigration frequencies of free-tachyzoites or tachyzoite-infected-DC across polarized BeWo monolayers on transwell filters. (**A**) *T. gondii* (Tg-RH-LDM and Tg-ME49-PTG strains) and (**B**) *N. caninum* (Nc-1, Nc-Liverpool, Nc-SweB1, Nc-Spain 3H, Nc-Spain 4H, Nc-Spain 6, Nc-Spain 7 and Nc-Spain 9 isolates). Quantification was performed by counting plaques formed in the lower chamber HFF monolayer. Box-plot and whiskers graph represents the lower, upper quartiles, median and minimum–maximum of number of migrated parasites values (×10^−3^) of the replicates from at least three independent experiments. The black bars indicate *T. gondii* and the white bars indicate *N. caninum*. Asterisks indicate, for each isolate, statistically significant differences between the transmigration frequencies of infected-DC and free-tachyzoites (P<0.05; *U*-Mann Whitney). **C**. Ratio of the median transmigration frequencies across polarized BeWo monolayers of tachyzoite-infected-DC/free-tachyzoites. Extracellular tachyzoites of Tg-RH-LDM and Nc-Liverpool, Nc-Spain 6 and Nc-Spain 9 exhibited a potent transmigratory capacity and thus a relatively lower dependency on DC-mediated transmigration. In contrast, Tg-ME49-PTG and Nc-Spain 4H, Nc-Spain 7, Nc-Spain 3H, Nc-1 and SweB1 showed a preferential dependency on DC-mediated transmigration with variability among isolates. The black bars indicate *T. gondii* and the white bars indicate *N. caninum*.

To investigate the ability of the tachyzoite stages from different isolates to cross biological barriers, e.g. the placenta, and to determine whether infected DC could promote the passage of parasites across cellular barriers, an *in vitro* system with polarized trophoblast-like BeWo cell monolayers was established. For *T. gondii*, extracellular type I tachyzoites (free Tg-RH-LDM) displayed a higher transmigration frequency compared to extracellular type II tachyzoites (free Tg-ME49-PTG) (P<0.05; *U*-Mann Whitney test) (Figure 2A). Interestingly, DC promoted the transmigration of type II tachyzoites (Tg-ME49-PTG infected-DC *versus* free Tg-ME49-PTG; P<0.05; *U*-Mann Whitney test) (Figure 2A). These findings are consistent with the notion that *T. gondii* type I parasites preferentially disseminate extracellularly [6] whereas type II parasites preferentially exploit the shuttling-function of DC [8].

To determine if this phenotypic diversity was conserved among other apicomplexan parasites, we studied the ability of different *N. caninum* isolates to cross polarized BeWo cell monolayers. Extracellular tachyzoites from all *N. caninum* isolates tested managed transmigration across the BeWo monolayers, with a wide range of transmigration frequencies among isolates isolates (Figure 2B) (P<0.05, Kruskal-Wallis *H* test). The highest transmigration frequencies were observed for Nc-Liverpool, Nc-Spain 6 and Nc-Spain 9. In parallel, the isolates were allowed to infect DC, extracellular parasites were removed, and passage of parasites across the monolayers was assessed. The frequency of transmigration for infected DC significantly varied between isolates (P<0.05; Kruskal-Wallis *H* test). DC infected with the isolates Nc-Spain 4H and Nc-Spain 7 exhibited the highest transmigration frequencies. Interestingly, most of the isolates previously shown to induce the highest frequencies of migration in DC (Figure 1B), also exhibited a significantly higher efficiency at crossing the BeWo cell barrier (Figure 2B, Nc-Spain 4H, Nc-Spain 7, Nc-Spain 3H and Nc-1: infected-DC *versus* free-tachyzoites; P<0.05; *U*-Mann Whitney test).

Quantification of transmigrated parasites by plaquing assays and real-time PCR yielded similar results, indicating high viability of transmigrated parasites (data not shown). No passage of the fluid-phase marker FITC-dextran or a decrease in the transcellular electrical resistance (TCER) were detected during or after the transmigration assay, indicating that the polarization of the BeWo monolayer was not disrupted [37].

*T. gondii* type II (Tg-ME49-PTG), *N. caninum* isolates Nc-Spain 4H, Nc-Spain 7, Nc-Spain 3H, Nc-1 and Nc-SweB1 exhibited a relatively higher dependency on DC-mediated transmigration for efficient translocation across polarized cellular monolayers *in vitro* (Figure 2C). In contrast, *T. gondii* type I (Tg-RH-LDM), *N. caninum* isolates Nc-Liverpool, Nc-Spain 6 and Nc-Spain 9 exhibited a relatively higher dependency on extracellular tachyzoite transmigration compared to other isolates (Figure 2C). We conclude that the preferential pathway for translocation across BeWo monolayers varies among isolates.

### *N. caninum* isolates exhibit differences in gliding motility

**Figure 3:**
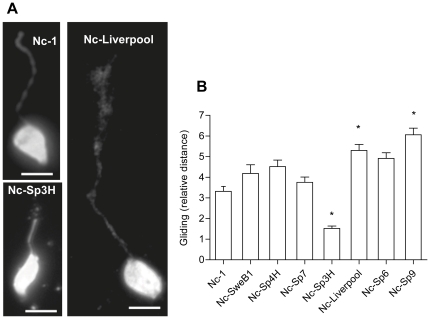
Gliding motility by *N. caninum* tachyzoites. **A**. *N. caninum* deposited surface membrane trails during gliding on a solid substrate. Trails were visualized by staining with anti-SAG1 mAb. Representative micrographs of Nc-1, Nc-Spain 3H and Nc-Liverpool trails are shown. Scale bar: 5 µm. **B**. *N. caninum* isolates exhibit significant differences in gliding motility (P<0.0001; one-way ANOVA test), being highest for Nc-Spain 9 (Nc-Spain 9 *versus* Nc-1, Nc-SweB1, Nc-Spain 3H and Nc-Spain 7; P<0.05-0.0001; Bonferroni's post-test), and lowest for Nc-Spain 3H (Nc-Spain 3H *versus* all isolates; P<0.05-0.0001; Bonferroni's post-test). The mean length of trails formed by Nc-Liverpool was also superior to that of Nc-1 (P<0.05; Bonferroni's post-test). Trails were measured as relative parasite body lengths. Ten to fifteen trails were measured per strain per experiment. Mean (±SEM) of the determinations from three independent experiments is represented. Asterisks indicate isolates which showed significant differences.

The tachyzoite stage of apicomplexan parasites lacks cilia or flagella and it's mode of locomotion, termed gliding motility, relies on the actin-myosin motor of the parasite [5]. To investigate whether the differences in transmigration of extracellular tachyzoites observed between *N. caninum* isolates were linked to variations in locomotion, the gliding motility of the parasites was assessed [6]. Gliding distances were significantly different between isolates (P<0.0001; one-way ANOVA test) (Figure 3A&B), being highest for Nc-Spain 9 (Nc-Spain 9 *versus* Nc-1, Nc-SweB1, Nc-Spain 3H and Nc-Spain 7; P<0.05-0.0001; Bonferroni's post-test), and lowest for Nc-Spain 3H (Nc-Spain 3H *versus* all isolates; P<0.05-0.0001; Bonferroni's post-test). The mean length of trails formed by Nc-Liverpool was significantly superior to that of Nc-1 (P<0.05; Bonferroni's post-test). These data indicate that *N. caninum* isolates exhibit differences in gliding motility.

### Adoptive transfer of tachyzoite-infected DC results in increased parasitic loads in mice

**Figure 4:**
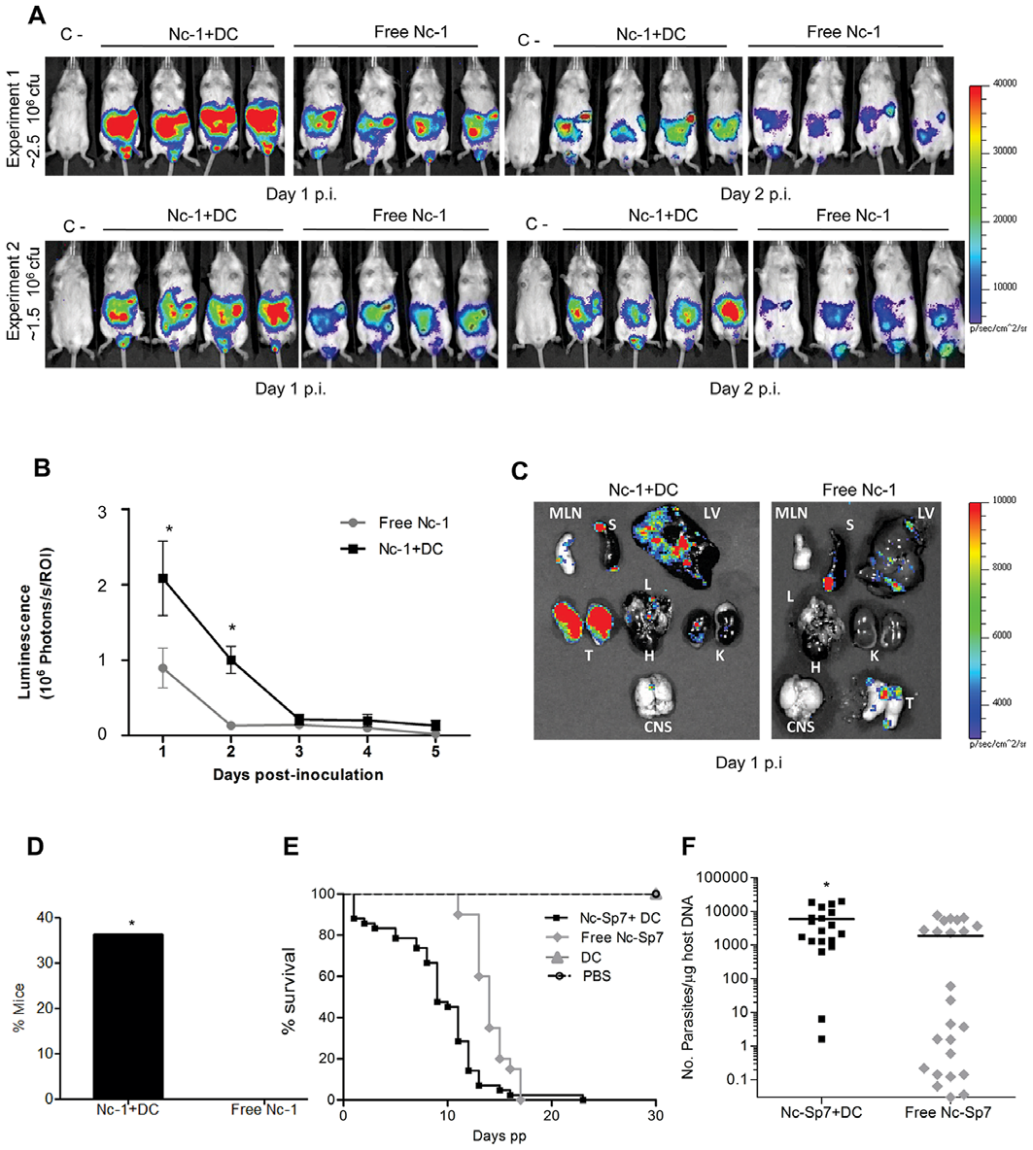
Adoptive transfer of *N. caninum* tachyzoite-infected DC in mice and parasite transmission to offspring during pregnancy. **A**. Kinetics of dissemination of *N. caninum* in BALB/c mice inoculated i.p. with ∼2.5×10^6^ cfu (experiment 1) or ∼1.5×10^6^ cfu (experiment 2) free Nc-1*Luc* tachyzoites or Nc-1*Luc*-infected DC. Progression of *N. caninum* infection and parasite biomass was assessed by bioluminescence imaging (BLI) using the IVIS Spectrum imaging system. Photon emission (photons s^−1^ cm^−2^) was assessed for 180 s 10–12 min after i.p. injection of d-luciferin. Images are from two experiments with four mice per group on days 1 and 2 p.i. Colour scales indicate photon emission (photons s^−1^ cm^−2^) during 180 s exposure time. **B**. Total photon emission analysis of BALB/c mice inoculated i.p. with free Nc-1*luc* tachyzoites or Nc-1*Luc*-infected DC on days 1–5 after inoculation. There was a dramatic increase in the parasite burden in mice inoculated with Nc-1*Luc*-infected DC compared to free Nc-1*Luc* on days 1 and 2 p.i. (P<0.05; Student's *t* test). Compiled data are from two independent experiments (day 1 p.i. n = 11; day 2–5 p.i. n = 8). **C**. Imaging of infected organs *ex vivo*. Organs were dissected on day 1 p.i from mice infected with free Nc-1*Luc* tachyzoites or Nc-1*Luc* -infected DC, respectively. Parasite load was significantly higher in the Nc1*Luc*-infected DC group. Signals were observed in images of the testis (T), liver (LV), spleen (S), mesenteric lymph nodes (MLN), lung (L), kidney (K) and brain (CNS). No signal was detected in the heart (H). **D**. Detection of *N. caninum* DNA in mouse brains on day 1 p.i. by real-time PCR as indicated under Materials and Methods. Y-axis indicates percentage of mice with positive PCR for mice infected with Nc-1*Luc*-infected DC or free Nc-1*Luc* tachyzoites, respectively (n = 11; positive 4/11 *versus* 0/11; P<0.05, Fisher *F* test). **E**. Kaplan–Meier survival curves for neonates born to dams inoculated with ∼2.5×10^6^ cfu of free Nc-Spain 7 tachyzoites or Nc-Spain 7-infected DC, 5×10^6^ non-infected DC or PBS buffer. The curves represent the percentage of animals surviving over a period of 30 days post-partum (pp). Vertical steps downward correspond to days pp when a mouse died or was sacrificed. Symbols indicate censored observations. The number of dead mice was registered daily, and the median survival time of the Nc-Spain 7-infected DC group was significantly shorter than that of the free Nc-Spain 7-infected group (P<0.001, Log-rank test). Compiled data are from two independent experiments (n = 7–14 pregnant mice per group). **F**. Parasite loads in brain from offspring quantified by real-time PCR and expressed in terms of number of parasites per µg of host DNA. Pups born to mice inoculated with Nc-Spain 7-infected-DC displayed significant higher parasite loads than the free Nc-Spain 7-infected group (P<0.01; Student's *t* test). The data are represented as individual points and horizontal lines correspond to the mean value. Compiled data are from two independent experiments.

To assess the potential impact of infected DC during *in vivo* infections, spatio-temporal analysis of the progression of infection in mice was performed by bioluminescence imaging [34] using a luciferase-expressing line (Nc-1*Luc*). Parasitic loads were assessed in mice inoculated with free-tachyzoites or with tachyzoite-infected-DC i.p. [8]. Interestingly, analysis of total photonic emissions indicated significantly higher parasitic loads in mice inoculated with Nc-1*Luc* infected-DC compared to mice inoculated with free tachyzoites on days 1 and 2 p.i. (P<0.05; Student's *t* test) (Figure 4A&B). This finding was confirmed when organs were extracted, with high photonic emissions in testis, liver, spleen and mesenteric lymph nodes (Fig. 4C). Emissions were lower in lungs and kidneys and undetectable or barely detectable in the heart and brain respectively. Interestingly, bioluminescence signal in the CNS was observed in only one mouse inoculated with infected-DC. Plaquing assays performed on testis and CNS confirmed the presence of viable parasites (data not shown). As parasite loads are normally low in CNS and photonic emissions can be impaired by the tissue [34], two sets of experiments were performed in order to corroborate that parasites reached CNS earlier in mice inoculated with infected-DC. On day 1 p.i., parasite DNA was only detected in the brain from mice inoculated with tachyzoite infected-DC (Fig. 4D). Taken together, these results show that adoptively transferred *N. caninum* infected-DC mediate a rapid dissemination of *N. caninum* tachyzoites in mice leading to a heavier parasite burden in vital organs and an earlier parasite presence in the CNS.

### Impact of adoptive transfer of tachyzoite-infected DC on congenital infection

A well-established pregnant BALB/c mouse model [17], [35] was used to test whether adoptive transfer of infected-DC could promote *N. caninum* vertical transmission. This model ensures a high transmission rate of *N. caninum* to the offspring after inoculation to dams at second trimester of gestation [38]. We used Nc-Spain 7, one of the isolates that induced a potent hypermigratory phenotype in DC and previously was shown to exhibit a high vertical transmission rate in the pregnant-mouse model [17]. A significantly shorter median survival time was observed in neonates from the infected-DC group (9 days) *versus* free tachyzoites (14 days) (P<0.001; Log-rank test) (Figure 4E). In order to test whether the differences in neonatal mortality observed in infected-DC group was due to a higher parasite congenital transmission, *N. caninum* loads in CNS from pups were determined. Pups born to mice inoculated with infected-DC displayed significantly higher parasite loads in brain (P<0.01; Student's *t* test) (Figure 4F). No significant differences in fertility (P>0.05, χ^2^ test), stillborn rates (P>0.05, Fisher *F* test) and litter size (P>0.05; one-way ANOVA) were observed among groups (data not shown). Altogether, these data suggest that inoculation of tachyzoite infected-DC favored the parasite transmission to offspring during pregnancy.

### Correlation analyses of migratory phenotypes, vertical transmission and neonatal mortality

**Figure 5:**
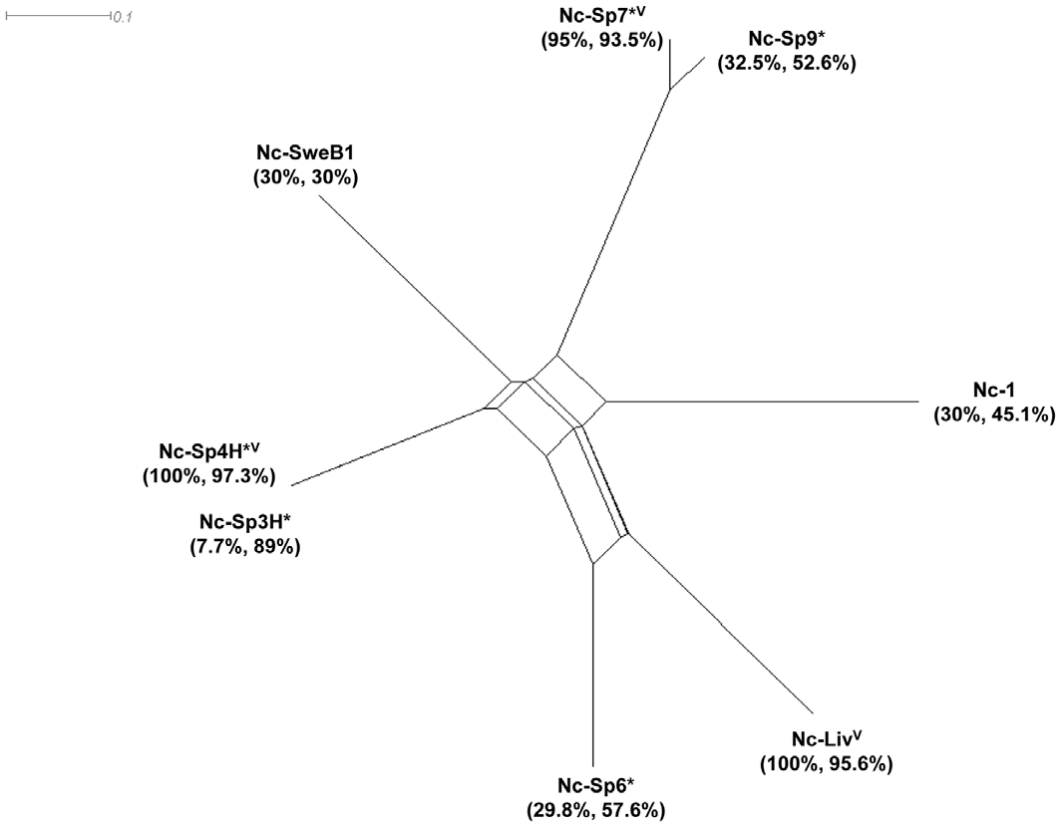
Characteristics of *N. caninum* isolates. NeighborNet phylogenetic network for the *N. ca*ninum isolates included in this study was based on multilocus genotypes determined by 9 microsatelite markers (MS4, MS5, MS6a, MS6b, MS7, MS8, MS10, MS12 and MS21) [18], [22]. Phylogenetic network analysis was developed using the shoftware SplitsTree4 (v 4.11.3). *N.caninum* isolates included the Spanish isolates: Nc-Spain 3H, Nc-Spain 4H, Nc-Spain 6, Nc-Spain 7 and Nc-Spain 9, which were obtained from asymptomatic calves [22]. Nc-1 was obtained from a clinically affected dog in the United States [19], Nc-Liverpool from a clinically affected dog in the United Kingdom [20] and Nc-SweB1 from a stillborn calf in Sweden [21]. The asterisk (*) indicates isolates obtained from asymptomatic animals. The percentages represent neonatal mortality and vertical transmission rates, respectively. The rates were determined in previous studies using a pregnant BALB/c mouse model [16 and unpublished data]. The letter “V” indicates highly virulent isolates according to the significant differences found in neonatal mortality. The rest of the isolates could be considered as lowly/moderately virulent [16].

To investigate potential associations between the *in vitro* and *in vivo* parameters evaluated in this study, a correlation analysis between the different phenotypes and strain characteristics was performed. A significant correlation was found between transmigration of infected DC across Transwell membranes and across polarized BeWo cell monolayers (ρ = 0.7; P<0.05, Spearman correlation test) (Fig. 1B and 2B). Additionally, a significant correlation was found between the frequency of transmigration across BeWo monolayers by extracellular tachyzoites and the gliding distance (ρ = 0.7; P<0.05) (Fig. 2B and 3B). In contrast, an inverse correlation was observed between the frequency of extracellular transmigration by tachyzoites and the frequency of transmigration by infected DC, but the result was not significant (ρ = −0.64; P>0.05). Altogether, this indicates that while some isolates preferentially rely on extracellular transmigration, other isolates rely on transportation by infected host-DC for passage across cellular barriers *in vitro* (Fig. 2C). In addition, results suggest that isolates with high extracellular transmigration capacity also would exhibit superior gliding motility. Next, the *in vitro* migratory phenotypes of the different isolates were compared to *in vivo* features (mouse neonatal mortality and vertical transmission rates) [17] (Fig. 5). No significant correlations could be discerned between these features, although an association close to significance was found between vertical transmission in mice and transmigration of infected DC (ρ = 0.7; P = 0.07).

## Discussion

The processes leading to systemic dissemination and to establishment of infections by apicomplexan parasites remain poorly characterized. Using *in vitro* systems that mimic biological barriers *in vivo*, this study establishes that tachyzoites from *N. caninum* are able to transmigrate across restrictive cellular monolayers (1) powered via their own active gliding motility and (2) by transportation in infected DC. The migratory pathways utilized by *N. caninum* are directly relevant to dissemination-related acute pathology, e.g., transmission of the parasite to the developing fetus.

During natural infections, the parasite's ability to rapidly cross epithelial barriers and reach the circulation likely represents an important component of dissemination, particularly to sites of immune privilege, i.e. the CNS and the developing fetus. Our findings demonstrate that active motility is not only used for invasion of host cells but also provides *N. caninum* with an effective mechanism of dissemination in its microenvironment within tissues. Moreover, the data indicates important differences in the ability to penetrate tissues among isolates. Furthermore, tachyzoite-infected DC exhibited a potent migratory phenotype that facilitated translocation of parasites across polarized cell monolayers. Interestingly, while all isolates tested exhibited this trait, the frequency of induction of DC migration varied between isolates. As shown for *T. gondii* [8], isolate-specific differences in migratory induction of DC were not explained by variations in infection, replication rates of parasites or by lysis of host cells, Furthermore, transmigration assays were performed during the lag phase that follows infection of host cells and that is characterized by slow replication of tachyzoites [18]. Altogether, this indicates that infected DC may contribute to parasite propagation in an isolate-specific manner.

The utilization of two complementary migratory pathways, direct transmigration by extracellular tachyzoites and transportation in infected DC or other leukocytes (Trojan horse), has important conceptual implications for how we consider passage to the fetus and to the CNS. The finding that all isolates tested exhibited both migratory traits indicates an evolutionary conservation of these phenotypes and is indicative of an underlying genetic control by the parasite [8]. Recently, we described that the related apicomplexan parasite *T. gondii* makes use of similar strategies for dissemination [6], [7], [8]. Thus, the evolutionary conservation of these traits may be extended across the apicomplexan family. Also, for *T. gondii*, an isolate-specific migration strategy was linked to the parasite genotype and virulence. While extracellular tachyzoites of genotype I exhibit a potent transmigratory ability in epithelial cell monolayers, this trait is significantly reduced in genotype II and genotype III strains [6]. In contrast, genotype II and genotype III strains induce a potent hypermigratory phenotype in DC *in vitro* and adoptive transfer of infected DC in mice led to substantially increased parasite loads in the circulation for type II and III parasites, but only a slight increase for type I parasites [8]. In the present study, a placenta *in vitro* model for passage of *Toxoplasma*-infected DC and extracellular tachyzoites was established. Interestingly, transmigration efficiency was similar for extracellular type I-tachyzoites and type II-infected DC. This suggests that the usage of different migration strategies or combined migratory strategies among isolates may also ultimately lead to comparable transplacental transmission. A similar isolate-specific migratory pattern was observed for *Neospora*, with strains exhibiting migratory traits similar to *Toxoplasma* type II strains or *Toxoplasma* type I strains in mice. Rather than mutually exclusive, the utilization of both pathways could be complementary *in vivo* [9]. It is conceivable that efficient transportation of parasites by infected leukocytes (Trojan horse) may locate parasites in target organs (e.g. the placenta and the CNS) and that passage across cellular barriers may be facilitated by the parasite motility-driven transmigration (e.g. placenta and blood brain barrier) or by assisted transmigration in infected leukocytes (e.g. blood brain barrier). In line with this, recent studies suggest that during the first days of infection, *N. caninum*-derived molecules attract monocytic cells to the sites of infection, thereby facilitating initial parasite invasion and proliferation [39]. Altogether, our data indicates that *N. caninum*, in a similar fashion to *T. gondii*, can utilize a combination of migratory strategies, which may benefit passage across restrictive biological barriers.

Here, we used *N. caninum* isolates obtained from symptomatic and asymptomatic hosts. These isolates have shown differences in virulence and in vertical transmission in mouse models ([17] and unpublished data). In previous studies, isolates yielding high neonatal mortality in mice were classified as virulent [17]. To date, a classification of virulent and non-virulent lines on a genetic basis is not evident for *N. caninum* [19], [23] (Fig. 5). This study did not find a clear association between *in vitro* migratory phenotypes, virulence *in vivo* and genetic characterizations. This could be due to the fact that parasite virulence traits are complex and factors such as host susceptibility to infection need to be considered. Moreover, a limited number of strains that are available in the field were tested. In terms of dissemination *in vivo* isolates with low virulence could have a lower efficiency at crossing biological barriers (either powered via their own active gliding motility or by transportation in infected leukocytes), leading to lower parasitic loads in the brain or the fetus.

Importantly, adoptive transfer of *Neospora*-infected DC accentuated vertical transmission of the parasite to the fetus and increased parasitic loads in the CNS. In a recent report, *N. caninum* tachyzoites were observed in close association with splenic DC shortly after intraperitoneal challenge [40]. Thus, transportation of parasites in infected DC could have implications for vertical transmission and peripheral dissemination, e.g. to the CNS. To address whether adoptive transfer of infected-DC could promote *N. caninum* dissemination *in vivo*, two of the isolates which induced a potent hypermigratory phenotype in DC *in vitro* (i.e. Nc-1 and Nc-Spain 7) were tested in the non-pregnant and pregnant mouse models. Both models share similar requirements for parasites, or parasite-infected DC, to actively cross biological barriers such as endothelial layers to reach the lymphatic system, brain or placenta. Importantly, adoptive transfer of *Neospora*-infected DC in mice mediated a rapid dissemination of tachyzoites leading to a higher parasite tissue burdens and an earlier parasite presence in the CNS. We also observed that pups from dams inoculated with Nc-Spain 7 infected-DC exhibited a shorter median survival time and higher brain parasite burdens. This is indicative that adoptive transfer of infected DC facilitated the passage of the Nc-Spain 7 across the placenta, increasing the quantity of parasites reaching the fetal tissues and consequently enhancing the parasite burdens and pathology, ultimately resulting in a shorter survival time. Further *in vivo* experiments are required to study differences in dissemination of *N. caninum* isolates and to investigate if some isolates disseminate more efficiently as free parasites or favor carrier cells.

In the present study, it cannot be excluded that the intracellular localization in DC or other leukocytes *per se* offers a safe intracellular niche. Yet, the induction of DC migration observed *in vitro* advocates for an additional migratory advantage. Similarly, this advantage was abolished when the motility of DC was inhibited *in vitro* and *in vivo* in the *T. gondii* model [7]. The present study does not address experimentally whether active migration of the infected DC across the placenta is necessary to yield higher parasitic loads in the offspring. Thus, it cannot be excluded that infected DC adhere to the placenta tissue to concentrate more parasites in the vasculature and that these parasites egress from DC to transmigrate across the placenta via the extracellular route. These possibilities are not mutually exclusive and await further investigation, specifically in bovine, where parasites must cross five cellular layers in the placenta in order to reach the fetus. Moreover, inflammatory responses during infection in pregnancy alter the placenta barrier function, including apoptosis of placental cells and transfer of maternal cells [41]. An accumulation of monocytic cells in the human placenta has been observed during *P. falciparum* infection [42]. Also, *in vitro* studies have shown that activated monocytic cells infected by *T. gondii* adhere to BeWo trophoblast cell monolayers, thereby facilitating infection by the parasite [43]. In addition, there are likely to be additional key determinants of parasite migration that allow transfer across the placenta, a tissue that does not normally support leukocyte trafficking.

Mounting evidence suggest that infection of leukocytes, e.g. DC and macrophages, may have modulatory effects on immune cell activation and cytokine secretion during *N. caninum* infection [12], [13] and during infections by other intracellular parasites [44]. Thus, in the host-parasite interplay, stimulatory and down-modulatory responses induced by the parasite have to be reconciled with the parasites need for dissemination. In this context, the present study reveals that *N. caninum* exploits the natural cell trafficking pathways in the host to cross cellular barriers and disseminate to deep tissues, likely using strategies reminiscent of leukocyte extravasation or by using leukocytes as Trojan horses. Also, our findings show that active motility is not only used for cell invasion but also may provide *N. caninum* with an effective mechanism of dissemination in its microenvironment within tissues. The elucidation of the migratory pathways utilized by *N. caninum* may have important conceptual implications for how we consider therapeutic and prophylactic approaches to hinder establishment of infection and vertical transmission to the fetus.
